# Chronic stress-induced apoptosis is mitigated by young mitochondria transplantation in the prefrontal cortex of aged rats 

**DOI:** 10.22038/IJBMS.2023.69551.15145

**Published:** 2023

**Authors:** Gonja Javani, Arshad Ghaffari-Nasab, Fereshteh Farajdokht, Gisou Mohaddes

**Affiliations:** 1 Drug Applied Research, Tabriz University of Medical Sciences, Tabriz, Iran; 2 Neurosciences Research Center, Tabriz University of Medical Sciences, Tabriz, Iran; 3 Department of Biomedical Education, California Health Sciences University, College of Osteopathic Medicine, Clovis, CA, USA

**Keywords:** Aging, Apoptosis, Chronic stress, Mitochondria, Oxidative stress, Rats

## Abstract

**Objective(s):**

Apoptosis is common and often comorbid with aging and stress-related mood disorders. Evidence suggests that fresh mitochondria could reverse age-related dysfunctions in organs, especially in the brain. Therefore, this study investigated the effect of young mitochondria administration on the apoptosis process in the prefrontal cortex (PFC) of aged rats exposed to chronic stress.

**Materials and Methods:**

Aged (22 months old) male rats were randomly assigned into four groups: aged control (AC), aged rats treated with young mitochondria (A+M), aged rats subjected to chronic stress for four weeks (A+St), and aged rats subjected to chronic stress and treated with young mitochondria (A+St+M). A+M and A+St+M groups received a single ICV injection (10 μl) of fresh mitochondria isolated from the brain of young rats for five minutes (2 µl/min). Finally, the levels of Malondialdehyde (MDA), Cytochrome c (Cyt c), Bax, Bcl-2, and Caspase-3 expression were investigated in the PFC.

**Results:**

Young mitochondria administration reduced neuronal apoptosis in the PFC, associated with down-regulation of MDA, Bax, and Caspase-3 and up-regulation of Bcl-2. Moreover, fresh mitochondria partially improved the chronic stress-induced mitochondrial dysfunction in aged rats, as indicated by reduced cytochrome c (Cyt c) release from the mitochondria.

**Conclusion:**

These results suggest mitotherapy could reverse cell viability and mitochondrial dysfunction-induced apoptosis in the PFC tissue of aged rats subjected to stressful stimuli.

## Introduction

Stress-related responses gradually change with advancing age, and it is evident that the effects of psychological stress are exacerbated during aging (1). In addition, aging is associated with loss of emotional and functional resilience to stressors in animal models and the human brain (2). The prefrontal cortex has shown more vulnerability to normal aging (3) and chronic stress, resulting in loss of neuron resilience in this brain area (2).

Mitochondria are essential organelles in regulating cellular homeostasis by involving bioenergetic changes, reactive oxygen species (ROS) generation, signal transduction, and apoptosis (4, 5). During aging, cellular characteristics of mitochondrial dysfunction, including impaired oxidative phosphorylation, increased ROS levels, impaired activity of metabolic enzymes, and changes in mitochondrial morphology and biogenesis, have been well addressed (6). As a well-established hallmark of aging, mitochondrial dysfunction contributes to the development of age-related pathological changes such as neurodegeneration (7). Emerging evidence indicated that impaired mitochondrial function plays an essential role in apoptosis and age-related physiological and pathophysiological processes (6, 8). A number of studies have also demonstrated that mitochondrial disturbances lead to an imbalance between oxidant and anti-oxidant factors and oxidative damage with advancing age (9). Besides, alterations in mitochondrial functions such as oxidative phosphorylation and apoptosis have been implicated in the development of chronic stress-induced mental illness (10). In this regard, oxidative stress markers such as MDA are affected by psychosocial stress, and their level is increased following mild stress in major depression (11). Several lines of evidence have demonstrated young mitochondrial anti-oxidant, anti-inflammatory, and anti-aging activities (12, 13). Moreover, mitochondria can reverse several aspects of age-related neuropathology at the molecular, functional, and cognitive levels in aged mice (12).

Apoptosis is conceived as an important process affecting neuronal and glial survival in aging (14) and contributes to mood changes, especially stress-related depression, which consequently reduces the effectiveness of antidepressant agents (15). It has been revealed that the number of apoptotic cells in the PFC is increased in stress-induced depression (16). Thus prefrontal cortical apoptosis could play a pathological role in the progression of stress-related depressive behaviors (16). Apoptosis is characterized by increased expression levels of apoptotic markers such as Bax and release of cytochrome-c from mitochondria (17). Cytochrome-c is also an important causative factor in the activated caspase cascade, which eventually causes caspase-3 to destroy DNA (18). 

Given the pivotal role of the mitochondria in aging and stress-related mood changes (19), the current study has been conducted to develop a novel protective strategy against chronic stress-induced pathophysiological changes in mitochondrial functions and apoptosis. We aimed to investigate the effect of the administration of young rats’ mitochondria on apoptosis in the PFC, a highly vulnerable brain area to aging and stress, in the stress-induced depression model of aged rats.

## Materials and Methods

***Ethics approval***

All animals were cared for according to the Guide for the Care and Use of Laboratory Animals of the National Institute of Health (8^th^ edition, Washington DC, National Academies Press (US); 2011) standards of the National Institutes of Health for Laboratory Animals Care and Use (NIH Publication No. 85-23, revised 1996). The procedures were approved by the Ethics Committee of Animal Research of Tabriz University of Medical Sciences [IR.TBZMED.VCR.REC.1400.003]. 

***Animals housing and experimental design***

**Figure 1:**
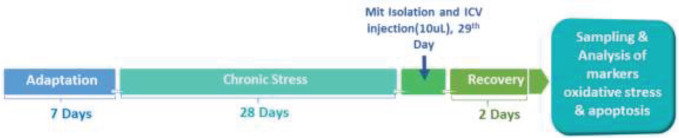
Timeline of the study procedures

Twenty-four (22 months old, weighing 450–550 g) and seven young (3 months old) male Wistar rats were randomly divided into four groups:  Aged control (AC), Aged rats treated with young mitochondria (A+M), Aged rats subjected to chronic mild stress (A+St), and Aged rats subjected to chronic mild stress and treated with young mitochondria (A+St+M). Each rat in A+M and A+St+M groups received a single ICV injection (10 μl) of fresh mitochondria isolated from the brain of young rats for five minutes (2 µl/min). The other two groups received the same volume of saline (vehicle). In stress-exposed groups, the standard light/dark cycle (12:12 hr) was changed in the course of the stress paradigm only. Additionally, food and water were available *ad libitum* except when food and/or water deprivation was applied as a stressor. The experimental design and timeline are presented in Figure 1.

***Chronic unpredictable mild stress protocol***

In stress groups, chronic mild unpredictable stressors were performed every day for four weeks. This protocol involves exposure to various stressors, including water and food deprivation (for 20 hr), tilt cage (45°, for 7 hr), intermittent white noise (85 dB, for 7 hr), strobe lighting (300 flashes/min, for 7 hr), and wet cage (150 ml water in bedding, for 17 hr) (20). 

***Mitochondrial isolation***

Young mitochondria were isolated from the brain of a young rat, according to a previous report (21). Briefly, the rat was euthanized, and the brain was dissected immediately. The brain was washed with cold PBS (0.01 M, pH 7.4), then cut into pieces. Homogenization of the brain samples was perfumed in cold isolation buffer. The homogenate was centrifuged at 1000 g for 5 min at 4 °C. The supernatant was collected and resuspended in the isolation buffer for another centrifugation at 3500 g for 10 min. The mitochondria pellet was washed with a second isolation buffer containing sucrose (70 mmol/L) and mannitol (210 mmol/L) in Tris/HCl (50 mmol/L) (pH=7.4) twice. Before injection, the number and concentration of extracted mitochondria were estimated under an optical microscope (Olympus, Tokyo, Japan) using a Bradford assay kit.

***Animal assignment and mitochondrial administration***

After labeling, the isolated mitochondria were administered into the right cerebral ventricle (10 µl in saline suspension) according to the following coordinates from Bregma: AP=−0.8 mm; ML=−1.5 mm; DV=−4 mm (21). Evidence of mitochondrial internalization into the brain cells was obtained by labeling with a mitochondrial-specific indicator, MitoTracker® Green FM (Cell signaling; 9074), according to the manufacturer’s protocol. Fourteen days after transplantation, the brain was sectioned using a freezing microtome, and tissue fluorescence was detected under a fluorescence microscope (AXIOM, BM-600 LED EPI, Germany).

***Tissue sampling***

After induction of deep anesthesia with ketamine and xylazine (80 and 10 mg/kg IP, respectively), the rats were sacrificed, and the whole brain tissue was dissected. Then PFC was carefully isolated on a cold plate according to Spijker *et al*. method (22) and stereotaxic atlas (23) and kept at −80 °C for molecular assessments. 

***Western blot analysis***

To determine the protein levels of caspase-3, Bax, Bcl-2, and cytochrome c, the prefrontal cortex of the right hemispheres was homogenized on ice in lysis buffer (500 µl, Tris-HCL, pH=8, 0.003 gr EDTA, 0.08 gr NaCl, 0.025 gr sodium deoxycholate, 0.01 gr SDS, one tablet protease inhibitor cocktail, 10 µl Triton NP40 (1%)) and was left for 20 min at 4 °C, then centrifuged (Eppendorf 5415 R) at 12,000×g for 10 min at 4 °C. The supernatant was stored at −20 °C. Proteins were separated by SDS-PAGE and transmitted onto PVDF membrane and then incubated for 2 hr at room temperature with primary antibodies against Caspase-3 (SANTA CRUZ, sc-7272), Bax (SANTA CRUZ, sc-7480), Bcl-2 (SANTA CRUZ, sc-492), cytochrome c (SANTA CRUZ, sc-13156), and β-actin (SANTA CRUZ, sc-47778) in the antibody buffer. Subsequently, a secondary antibody (donkey anti-goat; Santa Cruz, sc-2020) was used to incubate blots for 1 hr. All protein bands were normalized against β-actin protein, and the density of the bands was quantified using Image J software (3).

***Malondialdehyde (MDA) detection***

MDA level was determined in the PFC by Biocore Diagnostik (ZellBio) MDA assay Kit (Ulm GmbH,

Germany) according to the manufacturer’s protocol.

***Statistical analysis***

Data were expressed in terms of mean ± SEM. The differences between groups were assessed using two-way ANOVA and Tukey’s test for *post hoc* comparisons (*P*<0.05). All statistical analyses were performed with Prism 8 software (GraphPad, La Jolla, CA, USA). Of note, authors involved in data analysis were blinded to the experimental groups.

## Results

***Effect of young mitochondria administration on oxidative stress marker in the PFC***

**Figure 2:**
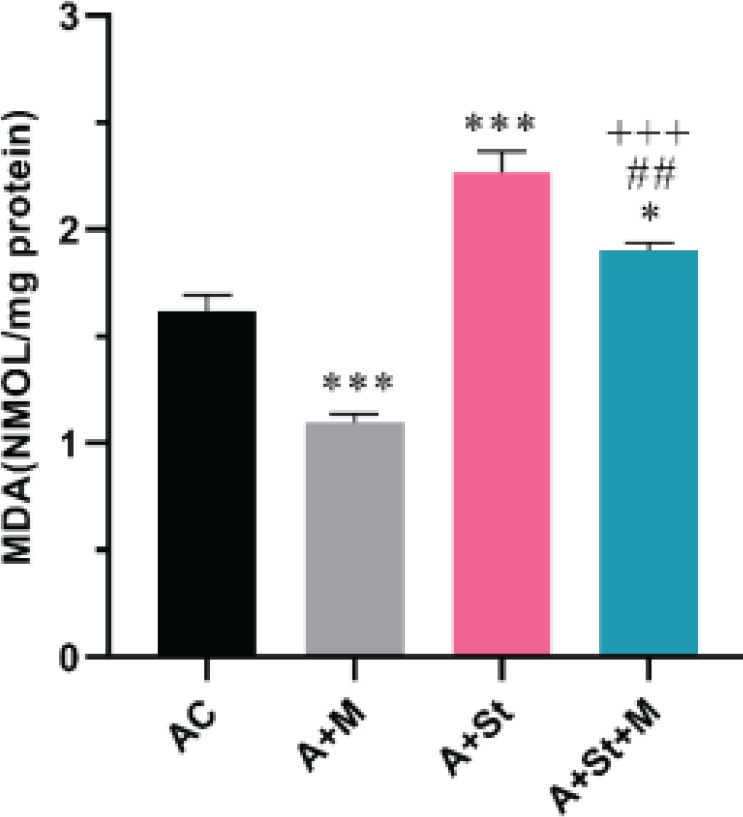
Effects of chronic stress and mitotherapy on MDA levels in the PFC of aged rats. Data are expressed as mean ± SEM (n=3). *P<0.05 and ***P<0.001 vs Aged control group, ## *P*<0.01 vs A+St group, +++*P*<0.001 vs A+M group. MDA, malondialdehyde; AC, Aged control group; A+M, Aged + mitochondria group; A+St, Aged + chronic stress group; A+St+M, Aged + chronic stress + mitochondria group

The two-way ANOVA of MDA levels showed a significant difference main effect of treatment [F (1, 20) =43.22, *P*<0.0001] and St [F (1, 20) =116.4, *P*<0.0001] but no main effect of treatment × stress interaction [F (1,20) =1.246, *P*=0.2775] between study groups (Figure 2). *Post-hoc* analysis showed that MDA levels significantly (*P*<0.001) decreased in the A+M group compared with the vehicle-received aged rats. Moreover, animals in the A+St group had significantly (*P*<0.001) higher MDA levels than the aged control group. However, treatment with young mitochondria in the A+St+M group significantly (*P*<0.01) decreased MDA levels compared with the A+St group. Moreover, MDA levels were significantly higher than in the aged control (*P*<0.05) and A+M (*P*<0.001) groups.

***Effect of mitotherapy on cytosolic cytochrome c levels in the PFC tissue***

**Figure 3:**
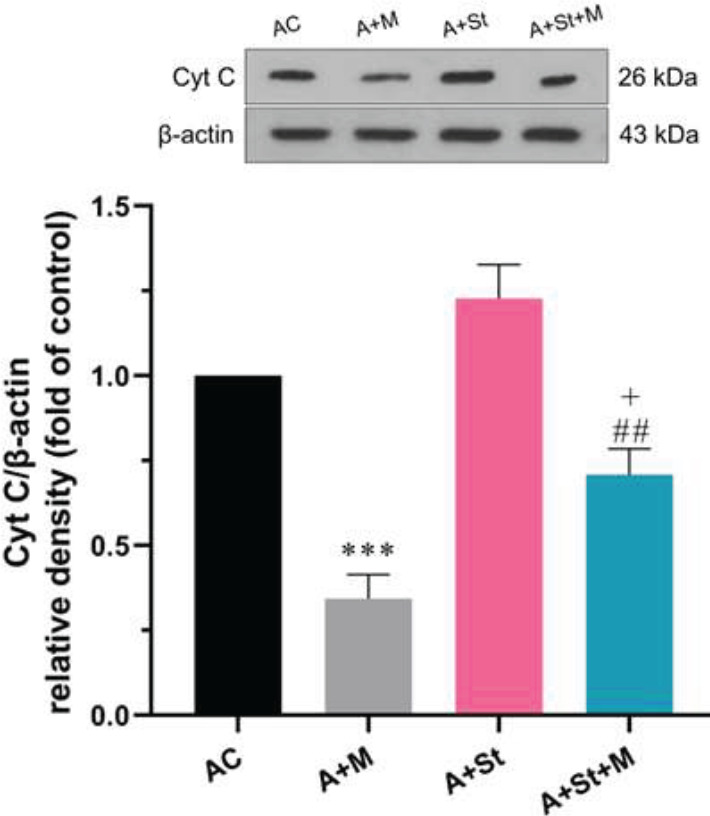
Effects of chronic stress and mitotherapy on Cyt c levels in the PFC of aged rats. Data are expressed as mean ± SEM (n=3). ***P<0.001 vs Aged control group, ##*P*<0.01 vs A+St group, + *P*<0.05 vs A+M group. Cyt c, cytochrome c; AC, Aged control group; A+M, Aged + mitochondria group; A+St, Aged + chronic stress group; A+St+M, Aged + chronic stress + mitochondria group

Based on the results of two-way ANOVA, there was a significant main effect of treatment [F (1, 8) =66.51, *P*<0.0001] and St [F (1, 8) =16.92, *P*=0.0034] but no main effect of treatment × stress interaction [F (1,8) =0.9164, *P*=0.3665] on the cytosolic Cyt c protein levels between study groups. As shown in Figure 3, mitochondria-treated animals exhibited a significant (*P*<0.001) decrease in the cytosolic Cyt c levels in the PFC compared with the aged control rats, indicating a decline in the release of Cyt c from the mitochondria. Young mitotherapy also significantly decreased the cytosolic Cyt c levels in the PFC of the A+St+M group compared with the A+St group (*P*<0.01). In contrast, the A+St+M group showed a significant (*P*<0.05) increase in Cyt c compared with the A+M group.

***Young mitochondria reduced apoptosis in the PFC of stress-exposed aged rats***

*Bax, Bcl-2, and caspase-3 expression levels*

**Figure 4:**
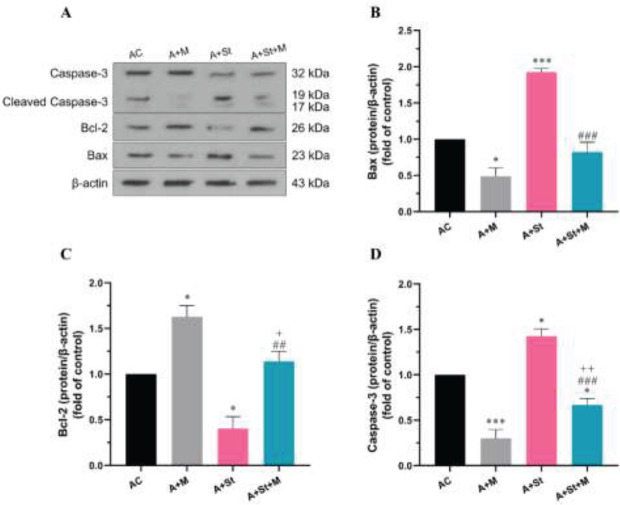
Effects of chronic stress and mitotherapy on Bax (A), Bcl-2 (B), and caspase-3 expression levels in the PFC of aged rats. Data are expressed as mean±SEM (n=3). *P<0.05 and ***P<0.001 vs Aged control group, ##*P*<0.01 and ###*P*<0.001 vs A+St group, +*P*<0.05 vs A+M group, and ++*P*<0.01 vs A+M group. AC, Aged control group; A+M, Aged + mitochondria group; A+St, Aged + chronic stress group; A+St+M, Aged + chronic stress + mitochondria group

As shown in Figure 4B, a two-way ANOVA showed a significant main effect of treatment [F (1, 8) =75.79, *P*<0.0001] and stress [F (1, 8) =46.21, *P*=0.0001] and a significant main effect of their interaction [F (1, 8) =10.04, *P*=0.0132] for Bax in the PFC tissue. Multiple comparisons indicated that mitotherapy significantly (*P*<0.05) decreased Bax in the A+M group compared with the aged control group. Moreover, chronic stress significantly (*P*<0.001) increased Bax compared with the aged control group. However, mitotherapy significantly (*P*<0.001) reduced Bax levels in the A+St+M group compared with the stress-subjected aged rats.

In addition, according to the results of two-way ANOVA of Bcl-2 protein levels (Figure 4C) using treatment and stress as factors, there was a significant main effect of treatment [F (1, 8) =42.50, *P*=0.0002] and stress [F (1, 8) =26.90, *P*=0.0008], but no significant main effect of their interaction [F (1, 8) =0.2477, *P*=0.6321] for Bcl-2 in the PFC. An intergroup comparison revealed that mitotherapy significantly (*P*<0.05) increased Bcl-2 expression in the A+M group compared with the aged control group. Notably, prefrontal protein levels of Bcl-2 significantly (*P*<0.05) decreased in the A+ St group compared with the AC group. Nevertheless, mitotherapy significantly (*P*<0.01) increased the protein expression of Bcl-2 compared with the A+St group. Besides, there was a significant (*P*<0.05) decrease in PFC Bcl-2 protein levels in the A+St+M group compared with the A+M groups.

The potential effect of young mitotherapy on the protein expression of caspase-3 (Figure 4C) was also investigated. The results of two-way ANOVA demonstrated significant main effects of treatment [F (1, 8) =107.1, *P*<0.0001] and Stress [F (1, 8) =32.09, *P*=0.0005], while no main effect of their interaction [F (1, 8) =0.1636, *P*=0.6965] among the experimental groups. The intergroup comparison revealed that young mitotherapy significantly (*P*<0.001) decreased caspase-3 expression in the A+M group compared with the aged control group. Notably, PFC protein levels of caspase-3 significantly (*P*<0.05) increased by chronic stress exposure compared with the aged control rats. Nevertheless, young mitotherapy significantly decreased protein expression of caspase-3 in A+St+M compared with the AC (*P*<0.05) and A+St (*P*<0.001) groups. Besides, there was a significant (*P*<0.01) decrease in PFC caspase-3 protein levels in the A+St+M group compared with the A+M.

## Discussion

The results of the present study demonstrated that the transplantation of young mitochondria ameliorated oxidative stress in the PFC of aged and chronic stress-exposed aged rats, as indicated by diminished MDA levels and reduced Cyt c release. Young mitochondria also markedly attenuated apoptosis markers in the PFC of aged and chronic stress-exposed aged groups, which was characterized by down-regulated expression levels of pro-apoptotic proteins, Bax and caspase-3, and up-regulated expression levels of anti-apoptotic protein Bcl-2. 

It has been revealed that stressful stimuli impair the detoxification capacity through an imbalance between oxidant and anti-oxidant factors, resulting in oxidative stress (24, 25). Oxidative stress may facilitate the development of physiological dysfunction and the incidence of physical and mental diseases (26). Evidence supports the notion that the brain is especially sensitive to oxidative stress, which contributes to the development of emotional stress (27). Brain activity and neuronal function are altered through oxidative stress, associated with behavioral changes and neuropsychiatric diseases such as depression and anxiety disorders (27, 28). Therefore, oxidative damage in the brain is considered a risk factor for neuropsychiatric disorders (27, 29). It has also been established that aged people are more susceptible to the deteriorating effects of oxidative stress, mainly leading to the accumulation of ROS-induced damages (30). Notably, a correlation between behavioral changes and cell oxidative status has been reported in human and animal models during aging (28, 31). Consistently, in the present study, chronic stress exposure increased MDA levels in the PFC of aged rats. However, treatment with young mitochondria alleviated chronic stress-induced oxidative damage. Anti-oxidative effects of mitochondria transplantation have been reported in several studies (32–34). In addition, the levels of NO and 3-nitrotyrosine (3-NT) as oxidative injury signatures have been reduced in the spinal cord injury models following mitochondrial transplantation (35). It has been suggested that the protective effects of injected mitochondria against oxidative stress are possibly mediated through alterations in mitochondrial dynamics (fusion and fission) (32).

It has been documented that mitochondria play a crucial role in regulating the apoptotic pathway and that mitochondrial malfunction contributes to the hyperactivation of apoptotic signaling with advancing age (36). This phenomenon is characterized by increased Cyt c release from mitochondria, enhanced Bax/Bcl-2 ratio, and activation of caspase-3 (37, 38). Cyt c release from mitochondria initiates the apoptosis cascade by augmentation of caspase activity (39, 40). In addition, Bax is a pro-apoptotic protein that exerts an essential role in mitochondria-dependent apoptotic cell death through induction of Cyt c release and reduced mitochondria membrane potential (41, 42). During aging, these mitochondrial dysfunctions and apoptotic pathway dysregulations play a pivotal role in the etiology of neurodegenerative disorders (43, 44). Consistent with these data, the current study pointed out mitochondrial dysfunction and exacerbated aging related-apoptosis in the PFC of aged rats subjected to chronic stress. Moreover, our results indicated that mitotherapy could modulate these alterations in the PFC region.

It has been accepted that chronic stress can aggravate apoptosis by enhancing the expression levels of caspase proteins in the cerebral cortex of aged rats (45). In addition, the involvement of the apoptosis process in the psychopathology of stress-related mood disorders is evident, especially in the PFC (16), a brain region involved in response to the stress stimuli and the pathogenesis of stress-induced mood disorders (46).

The administration of isolated mitochondria has recently been regarded as a potential therapeutic strategy for many diseases associated with mitochondrial dysfunction (21). Previous studies have also reported evidence that mitochondria transplantation exerts an anti-apoptotic effect in spinal cord injury and cardiac ischemia models (35, 47, 48). In these studies, healthy mitochondria treatment has been shown to increase the expression levels of Bcl-2 and reduce Bax and cleaved caspase-3. In addition, ICV injection of exogenous mitochondria attenuated oxidative stress and neuronal apoptosis and increased mitochondrial number and function in diabetic mice (49). Furthermore, mitochondrial transplantation increased cellular viability via mechanisms by which the apoptosis rate was suppressed in an oxygen-dependent manner (50). Based on the data, the anti-apoptotic effects of mitochondria administration could partially contribute to improving chronic stress-induced cellular and molecular changes in the PFC of old adults.

## Conclusion

This study highlighted the protective effects of mitotherapy against oxidative stress and apoptosis during aging and provided novel evidence that the injection of young mitochondria could alleviate aging and chronic stress-induced apoptotic changes in the PFC region by suppressing oxidative stress.

## Authors’ Contributions

GJ, AGN, FF, and GM designed the experiments; GJ and AGN performed experiments and collected data; GJ performed analysis and interpretation of results; FF and GJ discussed the results and strategy; GM supervised, directed, and managed the study; GJ, AGN, FF, and GM approved the final version to be published.

## Conflicts of Interest

The authors report there are no competing interests to declare.

## References

[1] Yamaguchi N, Nakajima N, Okada S, Yuri K (2016). Effects of aging on stress-related responses of serotonergic neurons in the dorsal raphe nucleus of male rats. Neurobiol Stress.

[2] McEwen BS, Morrison JH (2013). The brain on stress: Vulnerability and plasticity of the prefrontal cortex over the life course. Neuron.

[3] Ghaffari-Nasab A, Badalzadeh R, Mohaddes G, Alipour MR (2021). Young plasma administration mitigates depression-like behaviours in chronic mild stress-exposed aged rats by attenuating apoptosis in prefrontal cortex. Exp Physiol.

[4] Srivastava S (2016). Emerging therapeutic roles for NAD+ metabolism in mitochondrial and age-related disorders. Clin Transl Med.

[5] Wallace DC, Fan W, Procaccio V (2010). Mitochondrial energetics and therapeutics. Annu Rev Pathol.

[6] Sun N, Youle RJ, Finkel T (2016). The mitochondrial basis of aging. Mol Cell.

[7] Srivastava S (2017). The mitochondrial basis of aging and age-related disorders. Genes.

[8] Haas RH (2019). Mitochondrial dysfunction in aging and diseases of aging. Biology.

[9] Guo CY, Sun L, Chen XP, Zhang DS (2013). Oxidative stress, mitochondrial damage and neurodegenerative diseases. Neural Regen Res.

[10] Allen J, Romay-Tallon R, Brymer KJ, Caruncho HJ, Kalynchuk LE (2018). Mitochondria and mood: Mitochondrial dysfunction as a key player in the manifestation of depression. Front Neurosci.

[11] Bajpai A, Verma AK, Srivastava M, Srivastava R (2014). Oxidative stress and major depression. J Clin Diagn Res.

[12] Zhao Z, Yu Z, Hou Y, Zhang L, Fu A (2020). Improvement of cognitive and motor performance with mitotherapy in aged mice. Int J Biol Sci.

[13] Nascimento-Dos-santos G, De-Souza-ferreira E, Linden R, Galina A, Petrs-Silva H (2021). Mitotherapy: Unraveling a promising treatment for disorders of the central nervous system and other systemic conditions. Cells.

[14] Chi H, Chang H-Y, Sang T-K (2018). Neuronal cell death mechanisms in major neurodegenerative Diseases. Int J Mol Sci.

[15] Dygalo NN, Kalinina TS, Bulygina V V, Shishkina GT (2012). Increased expression of the anti-apoptotic protein bcl-xl in the brain is associated with resilience to stress-induced depression-like behavior. Cell Mol Neurobiol.

[16] Zhao Z, Zhang L, Guo XD, Cao LL, Xue TF, Zhao XJ (2017). Rosiglitazone exerts an anti-depressive effect in unpredictable chronic mild-stress-induced depressive mice by maintaining essential neuron autophagy and inhibiting excessive astrocytic apoptosis. Front Mol Neurosci.

[17] Eskes R, Antonsson B, Osen-Sand A, Montessuit S, Richter C, Sadoul R (1998). Bax-induced cytochrome c release from mitochondria is independent of the permeability transition pore but highly dependent on Mg2+ Ions. J Cell Biol.

[18] Hongmei Z (2012). Extrinsic and intrinsic apoptosis signal pathway review. Apoptosis and medicine.

[19] Reutzel M, Grewal R, Dilberger B, Silaidos C, Joppe A, Eckert GP (2020). Cerebral mitochondrial function and cognitive performance during aging: A longitudinal study in NMRI mice. Oxid Med Cell Longev.

[20] Ghaffari-Nasab A, Badalzadeh R, Mohaddes G, Javani G, Ebrahimi-kalan A, Alipour MR (2022). Young plasma induces antidepressant-like effects in aged rats subjected to chronic mild stress by suppressing indoleamine 2,3-dioxygenase enzyme and kynurenine pathway in the prefrontal cortex. Neurochem Res.

[21] Javani G, Babri S, Farajdokht F, Ghaffari-Nasab A, Mohaddes G (2022). Mitochondrial transplantation improves anxiety- and depression-like behaviors in aged stress-exposed rats. Mech Ageing Dev.

[22] Spijker S ( 2011). Dissection of Rodent Brain Regions. Neuromethods.

[23] Paxinos G, Franklin KBJ (2001). The mouse brain in stereotaxic coordinates: Hard cover edition.

[24] Liu J, Wang X, Mori A (1994). Immobilization stress-induced anti-oxidant defense changes in rat plasma: effect of treatment with reduced glutathione. Int J Biochem.

[25] Maes M, Galecki P, Chang YS, Berk M (2011). A review on the oxidative and nitrosative stress (O&NS) pathways in major depression and their possible contribution to the (neuro)degenerative processes in that illness. Prog Neuropsychopharmacol Biol Psychiatry.

[26] Kregel KC, Zhang HJ (2007). An integrated view of oxidative stress in aging: Basic mechanisms, functional effects, and pathological considerations. Am J Physiol - Regul Integr Comp Physiol.

[27] Bouayed J, Rammal H, Soulimani R (2009). Oxidative stress and anxiety Relationship and cellular pathways. Oxid Med Cell Longev.

[28] Bouayed J (2011). Relationship between oxidative stress and anxiety: Emerging role of anti-oxidants within therapeutic or preventive approaches. In: anxiety disorders. InTech.

[29] Bouayed J (2010). Polyphenols: A potential new strategy for the prevention and treatment of anxiety and depression. Curr Nutr Food Sci.

[30] Liguori I, Russo G, Curcio F, Bulli G, Aran L, Della-Morte D (2018). Oxidative stress, aging, and diseases. Clin Interv Aging.

[31] Michel TM, Frangou S, Thiemeyer D, Camara S, Jecel J, Nara K (2007). Evidence for oxidative stress in the frontal cortex in patients with recurrent depressive disorder--a postmortem study. Psychiatry Res.

[32] Lee JM, Hwang JW, Kim MJ, Jung SY, Kim KS, Ahn EH (2021). Mitochondrial transplantation modulates inflammation and apoptosis, alleviating tendinopathy both in vivo and in vitro. Anti-oxidants.

[33] Shi C, Guo H, Liu X (2021). Platelet mitochondria transplantation rescues hypoxia/reoxygenation-induced mitochondrial dysfunction and neuronal cell death involving the FUNDC2/PIP3/Akt/FOXO3a axis. Cell Transplant.

[34] Aharoni-Simon M, Ben-Yaakov K, Sharvit-Bader M, Raz D, Haim Y, Ghannam W (2022). Oxidative stress facilitates exogenous mitochondria internalization and survival in retinal ganglion precursor-like cells. Sci Rep.

[35] Lin M-W, Fang S-Y, Hsu J-YC, Huang C-Y, Lee P-H, Huang C-C (2022). Mitochondrial transplantation attenuates neural damage and improves locomotor function after traumatic spinal cord injury in rats. Front Neurosci.

[36] Tower J (2015). Programmed cell death in aging. Ageing Res Rev.

[37] Kwak H-B (2013). Effects of aging and exercise training on apoptosis in the heart. J Exerc Rehabil.

[38] Wang X, Bonventre J, Parrish A (2014). The aging kidney: Increased susceptibility to nephrotoxicity. Int J Mol Sci.

[39] Jeong SY, Seol DW (2008). The role of mitochondria in apoptosis. BMB Rep.

[40] Abu-Qare AW, Abou-Donia MB (2001). Biomarkers of apoptosis: Release of cytochrome c, activation of caspase-3, induction of 8-hydroxy-2’-deoxyguanosine, increased 3-nitrotyrosine, and alteration of p53 gene. J Toxicol Environ Health B Crit Rev.

[41] Nechushtan A, Smith CL, Lamensdorf I, Yoon SH, Youle RJ (2001). Bax and Bak coalesce into novel mitochondria-associated clusters during apoptosis. J Cell Biol.

[42] Acehan D, Jiang X, Morgan DG, Heuser JE, Wang X, Akey CW (2002). Three-dimensional structure of the apoptosome: Implications for assembly, procaspase-9 binding, and activation. Mol Cell.

[43] Ghavami S, Shojaei S, Yeganeh B, Ande SR, Jangamreddy JR, Mehrpour M (2014). Autophagy and apoptosis dysfunction in neurodegenerative disorders. Prog Neurobiol.

[44] Agostini M, Tucci P, Melino G (2011). Cell death pathology: Perspective for human diseases. Biochem Biophys Res Commun.

[45] Bachis A, Cruz MI, Nosheny RL, Mocchetti I (2008). Chronic unpredictable stress promotes neuronal apoptosis in the cerebral cortex. Neurosci Lett.

[46] Fan C, Song Q, Wang P, Li Y, Yang M, Yu SY (2018). Neuroprotective effects of ginsenoside-rg1 against depression-like behaviors via suppressing glial activation, synaptic deficits, and neuronal apoptosis in rats. Front Immunol.

[47] McCully JD, Cowan DB, Pacak CA, Toumpoulis IK, Dayalan H, Levitsky S (2009). Injection of isolated mitochondria during early reperfusion for cardioprotection. Am J Physiol Hear Circ Physiol.

[48] McCully JD, Cowan DB, Emani SM, del Nido PJ (2017). Mitochondrial transplantation: From animal models to clinical use in humans. Mitochondrion.

[49] Ma H, Jiang T, Tang W, Ma Z, Pu K, Xu F (2020). Transplantation of platelet-derived mitochondria alleviates cognitive impairment and mitochondrial dysfunction in db/db mice. Clin Sci.

[50] Xie Q, Zeng J, Zheng Y, Li T, Ren J, Chen K (2021). Mitochondrial transplantation attenuates cerebral ischemia-reperfusion injury: Possible involvement of mitochondrial component separation. Braun R, editor. Oxid Med Cell Longev.

